# Managing Anesthesia for a Patient With Von Willebrand Disease: A Case Report

**DOI:** 10.7759/cureus.90876

**Published:** 2025-08-24

**Authors:** Joshua Singavarapu, Galina Borodulina

**Affiliations:** 1 Anesthesiology, State University of New York Downstate Health Sciences University, Brooklyn, USA; 2 Anesthesiology, Northwell Health Staten Island University Hospital, Staten Island, USA

**Keywords:** pediatric anesthesiology, pediatric hematology, transexemic acid, von-willebrand disease, von-willebrand factor

## Abstract

Von Willebrand disease (vWD) is a common inherited bleeding disorder caused by a deficiency of von Willebrand factor (vWF), a glycoprotein involved in hemostasis. The two major roles of vWF are platelet adhesion and the stabilization of factor VIII in the coagulation cascade. The patient discussed in this case report was an eight-month-old male with diagnosed Type 1 vWD scheduled for circumcision due to phimosis. The preoperative evaluation showed no abnormalities. vWD was managed with the administration of a tranexamic acid bolus dose of 80 mg, followed by a continuous infusion throughout the case. The surgical course was uneventful; the patient maintained normal sinus rhythm and stable vital signs. Upon emergence, the patient had an episode of laryngospasm that was treated successfully with succinylcholine 20 mg. In the post-anesthesia care unit (PACU), the patient’s vital signs were stable, and there were no signs of postoperative hemorrhage or bleeding difficulties. It is important for anesthesiologists to learn from this case because it highlights the complexity and heightened attention required when managing pediatric patients with underlying coagulation disorders. This case demonstrates the potential for significant morbidity associated with inadequate perioperative care and the need for coordination among a multidisciplinary team.

## Introduction

Von Willebrand disease (vWD) is the most common inherited bleeding disorder, affecting about 1 in 10,000 individuals [1]. It is caused by a deficiency of von Willebrand factor (vWF), a glycoprotein involved in hemostasis. The two major roles of vWF are platelet adhesion at sites of damaged endothelium and the stabilization of factor VIII in the coagulation cascade [2]. Clinical symptoms of vWD are easy bruising, nosebleeds, and heavy menstrual bleeding. There are three main types of vWD, each varying in inheritance pattern and severity levels. Type 1 is the most common and has an autosomal dominant inheritance pattern with a mild clinical presentation and a quantitative defect of vWF. Type 2 is mostly autosomal dominant as well, with a qualitative defect of vWF and a usually mild presentation. Type 3 is the rarest subtype, being autosomal recessive, and is considered severe in presentation. Lab findings often show prolonged activated partial thromboplastin time (aPTT), and treatment consists of desmopressin for mild cases and vWF-containing factor VIII concentrates for severe bleeding [3]. 

From an anesthesiologist’s perspective, vWD is important to recognize because of the increased risks of perioperative bleeding. In the perioperative period, minor interventions such as peripheral IV insertion, airway manipulation, and mucosal instrumentation can cause excessive bleeding without proper management [4]. Management for these patients includes extensive history taking of bleeding disorders in the family and a review of specific labs such as von Willebrand factor antigen and activity, factor VIII levels, bleeding time, and PTT [5]. Postoperatively, patients should also be monitored for signs of bleeding, including wound hematomas and excessive drainage [6].

## Case presentation

The patient is an eight-month-old male diagnosed with mild Type 1 vWD. Family history was significant for the mother and maternal uncle, both of whom had the mild form of the disease. The patient had not experienced any bleeding or bruising episodes. He was scheduled for circumcision due to phimosis. Preoperative evaluation showed a healthy child with normal skin, heart, and lung examinations and a normal airway examination. Additional evaluation of coagulation levels showed low vWF activity, low vWF antigen, and low factor VIII. The anesthesiology team worked alongside the hematology team to create a thorough plan to minimize hemorrhagic complications. The patient was given Humate-P, a vWF-factor VIII concentrate, to optimize coagulation. He weighed 8 kg and had stable vital signs. He was at an appropriate stage of development for his age. The patient was classified as American Society of Anesthesiologists (ASA) Class II. The anesthetic plan was inhalation induction with a laryngeal mask airway (LMA).

Anesthesia was induced with sevoflurane 8% in 100% oxygen. A peripheral IV was placed, and 5 mcg of fentanyl and 30 mg of propofol were administered. Propofol was given to deepen anesthesia before surgical stimulation. An LMA size 2 was placed with good air exchange. An LMA was selected as it minimizes hemodynamic stress by reducing sympathetic stimulation and offers a less invasive approach that lowers the risk of airway trauma. Anesthesia was maintained with sevoflurane gas, and a penile block with bupivacaine 0.25% in an 8 mL solution was administered for postoperative pain management. vWD was managed with the administration of a tranexamic acid bolus dose of 80 mg, followed by a continuous infusion throughout the case. Tranexamic acid was used without desmopressin because circumcision is a high-fibrinolysis mucosal surgery. With tranexamic acid, fibrinolysis can be inhibited, whereas desmopressin only increases vWF and factor VIII levels, which helps form a clot but does not prevent it from breaking down. The patient was also given Humate-P to prevent surgical bleeding. Humate-P is a plasma-derived concentrate containing both vWF and factor VIII, used to treat and prevent bleeding episodes in patients with vWD or hemophilia A. During the case, maintenance of normothermia via a temperature probe, fluid management through an IV fluid bolus, and monitoring of coagulation parameters through a preoperative assessment of coagulation factors for baseline were performed. The surgical course was uneventful; the patient maintained normal sinus rhythm and stable vital signs. Upon emergence, the child had an episode of laryngospasm during removal of the LMA that was treated successfully with succinylcholine 20 mg. Succinylcholine is given during severe laryngospasm because it rapidly relaxes the laryngeal muscles, breaking the vocal cord closure within seconds. Its fast onset and short duration make it the most reliable rescue, as it quickly restores ventilation and oxygenation.

The patient was extubated successfully and taken to the post-anesthesia care unit (PACU) in stable condition. He was awake, at baseline mental status, and had minimal pain, which was measured by retraction and crying on palpation. In the PACU, the patient’s vital signs were stable, and there were no signs of postoperative hemorrhage or bleeding difficulties.

## Discussion

This case is important in highlighting perioperative management in pediatric patients with vWD. In this specific case, with a patient undergoing circumcision due to phimosis, there was an increased bleeding risk. From an anesthesiologist’s perspective, this case underscores the value of a comprehensive preoperative evaluation and collaboration [7]. Preoperatively, the anesthesiology team worked with the hematology team to create a thorough plan to minimize complications. Intraoperatively, careful maintenance of normothermia, appropriate fluid management, and vigilant monitoring of coagulation parameters were essential adjuncts that contributed to optimal patient outcomes. Additionally, as per a joint decision by the hematology and anesthesiology teams, tranexamic acid, an antifibrinolytic, was used to reduce perioperative bleeding. Careful management of IV fluids and maintenance of normothermia throughout the case helped optimize coagulation function during anesthesia [8]. Postoperatively, the patient also needed to be managed carefully for any signs of hemorrhage. In this case, there were no signs of hemorrhage and no complications.

Furthermore, patients with vWD have impaired hemostasis due to quantitative or qualitative defects in von Willebrand factor, significantly increasing their risk of perioperative bleeding. For anesthesiologists, this means careful consideration must be given to every aspect of patient management, from initial assessment and consultation with hematology to intraoperative monitoring and postoperative care. Thus, anesthesiologists play a pivotal role in facilitating communication across multidisciplinary teams, including surgeons, hematologists, and nursing staff, to ensure a unified approach to patient care. Specifically, anesthesiologists, to fully prepare for this operation, needed to delegate primary communication between teams to ensure a smooth surgery. Multidisciplinary communication was also essential for the surgical team in understanding the risk and complexity of the procedure, given the patient’s history. This case highlights that careful perioperative strategies, tailored specifically to the patient’s subtype of vWD and the surgical procedure, significantly decrease morbidity and improve outcomes. Ultimately, anesthesiologists should prioritize thorough preoperative evaluations, personalized anesthetic planning, and continuous perioperative vigilance for patients with vWD. Increased awareness and preparedness for managing vWD will enhance patient safety, reduce perioperative complications, and reinforce the critical role anesthesiologists play in comprehensive patient care.

## Conclusions

This case is presented to highlight the unique anesthetic challenges in managing pediatric patients with vWD undergoing surgery. Beyond reiterating standard recommendations, it demonstrates how individualized perioperative planning, such as selecting tranexamic acid and factor concentrate instead of routine desmopressin when its efficacy or safety is uncertain, can optimize hemostatic control. It also emphasizes the importance of anticipating airway complications, though unrelated to vWD, as this patient developed laryngospasm after LMA removal, which was rapidly and effectively treated with succinylcholine. The report adds to the literature by illustrating the integration of dynamic coagulation management, targeted pharmacologic choices, and prompt airway rescue into a cohesive, multidisciplinary plan. Applying these principles can help anesthesiologists minimize morbidity and improve safety in complex pediatric surgical cases.
